# Biodegradation of azo dyes by *Aspergillus flavus* and its bioremediation potential using seed germination efficiency

**DOI:** 10.1186/s12866-024-03703-9

**Published:** 2025-01-09

**Authors:** Amira M. Ghanaim, Omima M. El Mahdy, Heba I. Mohamed

**Affiliations:** Department of Biological and Geological Sciences, Faculty of Education, Ain Shams University, Cairo, 11341 Egypt

**Keywords:** Bromophenol blue, Congo red, Crystal violet, Decolorization, FT-IR, Malachite green, Phytotoxicity, *Zea mays*

## Abstract

The worldwide textile industry extensively uses azo dyes, which pose serious health and environmental risks. Effective cleanup is necessary but challenging. Developing bioremediation methods for textile effluents will improve color removal efficiency. The recent attention to effectively utilizing microbes to convert toxic industrial azo dyes into non-hazardous compounds has garnered significant attention. In the present study, four fungal strains—*Aspergillus flavus, Aspergillus terreus, Aspergillus niger*, and *Fusarium oxysporium*—were employed to screen for the degradation and detoxification of azo dyes including congo red, crystal violet, bromophenol blue, and malachite green. After eight days, *A. flavus* had degraded azo dyes at the maximum proportion. The maximum decolorization (%) was achieved at 50 mg/L of dye concentration, 8 days of incubation, pH 6, 30 °C temperature, sucrose as a carbon source, NaNO_3_ as a nitrogen source, Ca^+2^ as minerals, and using static culture. The efficient production of laccases, lignin peroxidase, and manganese peroxidase enzymes by *A. flavus* proved that the enzyme played a crucial role in decolorizing the harmful azo dyes. The Fourier Transform Infrared spectrometer (FT-IR) data validated the decolorization and degradation process brought on by absorption and biodegradation. Compared to control plants, the results of the phytotoxicity assay showed that the degraded product was less harmful to maize and common bean plant's growth and germination rates. As a result, the findings indicate that *A. flavus* is a viable option for remediating azo dyes. This aids in the biodegradation of azo dyes found in wastewater.

## Introduction

Chemicals are being used and released into the environment due to the world's rapid industrialization to meet the demands of the expanding population [1]. Many different types of dyes are utilized in the industrial processes of the food, plastic, textile sectors, and pharmaceutical industries [2]. An estimated 7 × 10^5^ tons of dye is produced annually, yielding 100,000 commercial dyes; nevertheless, a larger portion of this dye is released into water sources as waste during industrial processes [3]. There are various types of textile dyes, including basic, acidic, reactive, and direct dyes, all of which are essentially water-soluble. These types are naturally persistent and can cause significant harm to marine life by blocking the passage of light, slowing down photosynthesis, reducing oxygen levels, and ultimately leading to the death of organisms [4]. Azo dyes are resistant to light, moisture, and oxidants due to their intricate aromatic structures. This resistance comes from the presence of azo (-N = = N-) bonds connected to aromatic amines and benzene or naphthalene rings, which make them resistant to cleavage [5]. This is an attribute that the industry desires, but it poses a risk to the environment.

Synthetic dyes offer numerous benefits over natural dyes, including greater stability, a wider range of colors, lower production costs, and easier application [6]. These benefits have led to their use in various industries, including food, paper, plastic, textiles, rubber, cosmetics, pharmaceuticals, petroleum additives, and photography [7, 8]. Synthetic dyes are mostly used by the textile industry to color its fabrics. Due to the inefficiency of the dyeing process, which results in 200,000 tons of colored fluids flowing to water bodies annually, 10–15% of colored waters are lost to effluents in these procedures [9]. As previously mentioned, synthetic dyes are highly stable and pose a significant environmental risk because of their extended environmental persistence and poor biodegradability [10, 11]. Water containing artificial dyes reduces the amount of aquatic biodiversity by obstructing sunlight and causing issues for photosynthetic aquatic plants and algae [12]**.** Numerous artificial dyes are poisonous, carcinogenic, and mutagenic [13]. Furthermore, dyes have the potential to build up in sediments, particularly in areas where wastewater is discharged, and to disrupt the aquatic system's natural equilibrium. Contaminant leaching may have an impact on the groundwater system [14].

Various physical and chemical methods are available for removing dyes from wastewater [15]. The principal chemical techniques are photocatalysis, electrochemistry, Fenton's reaction, ozone, and hydrogen peroxide oxidation [16, 17]. In addition to their higher costs and operational challenges, chemical and physical treatments exhibit low efficacy, limited adaptability, susceptibility to interference from other wastewater constituents, and difficulties in managing created residues [18].

In recent decades, there have been suggestions to use biological techniques as a practical and efficient alternative to synthetic dyes in addressing the dye problem. These techniques rely on bioremediation, which involves using microorganisms to catalyze the complete breakdown or conversion of toxic substances into less harmful or environmentally friendly forms [19]. Numerous studies have confirmed the significant potential of various bacterial, fungal, and algal species in dye removal [20–22]. Dye removal can be achieved through biomass adsorption, biodegradation, or both methods. Dye biotransformation can lead to complete mineralization or the production of less harmful byproducts [23].

Fungi can transform aromatic compounds such as pesticides, lignin, and polycyclic aromatic hydrocarbons (PAH) through an extracellular enzymatic system. Currently, procedures for fungus decolorization are gaining significant attention [24]. Biodegradation and biotransformation enzymes can be obtained from fungal biomass and used as a sorbent. The biosorption process is characterized by its speed, effectiveness, and adaptability to various types of textile effluents [25–27]. *Aspergillus niger* and *Fusarium oxysporium* are two examples of non-ligninolytic fungi that exhibit biosorption; their dead biomass can be utilized as an adsorbent [28, 29]. White rot fungus produces enzymes such as lignin peroxidase (LiP) and Mn-dependent peroxidase (MnP), and laccase (Lac), and is the best studied. Its non-specific enzymatic activity enables it to break down a wide range of aromatic molecules and mediated dye decolorization [30–32]. Additionally, it was mentioned that non-ligninolytic enzymes might be involved in the breakdown of dyes like triphenylmethane crystal violet [30]. Dye was eliminated from tested strains' living biomass more successfully than their dead biomass [33]. The overall mechanism by which ligninolytic enzymes work involves the generation of free radicals, which set off a sequence of intricate events that ultimately cause the pigments to break [34]. The efficiency of decolorization is mostly dependent on the strain employed in the procedure, and the functional groups in the cell wall [35–40]. The sources and concentrations of carbon and nitrogen are crucial variables because they impact the ligninolytic enzyme synthesis. Numerous studies have been conducted on the impact of various carbon and nitrogen sources on the efficiency of decolorization [36, 37].

To determine the most effective fungus, we conducted a screening of the biodegradation capabilities of *Aspergillus flavus* AUMC8653, *Aspergillus niger* AUMC8669, *Aspergillus terreus* AUMC8625 and *Fusarium oxysporum* MT032355 on various azo dyes. Our goal was to maximize decolorization under different incubation conditions such as pH, temperature, carbon sources, nitrogen sources, and metal salts. We also aimed to study the enzymatic processes involved in breaking down azo dyes and the resulting degraded products. After treatment, we analyzed the fungal biomass using FT-IR to assess surface sorption. Finally, we conducted a phytotoxicity test using *Zea mays* and common bean seeds as the last step in the environmental risk assessment process.

## Materials and methods

### Chemicals and reagents

All chemical and azo dyes (congo red, crystal violet, bromophenol blue and malachite green) were purchased from Sigma-Aldrich Pvt. Ltd. Stock solutions of azo dyes (200 mg/L) were made by dissolving azo dyes into sterilized and distilled water, then filtered with a Whatman filter paper (No. 1) then kept in a refrigerator at 4 ± 1 °C until further used.

### Microorganism

*A. flavus* AUMC8653, *A. niger* AUMC8669, *A. terreus* AUMC8625 and *F. oxysporum* MT032355 were used in this study.

### Screening of microorganisms' efficiency

For the experiments, Czapek's Dox broth medium was used for the growth of *A. flavus, A. niger, A. terreus,* and *F. oxysporium*. Distilled water was used to dissolve the medium and 1% stock dye solution was added, and the medium was autoclaved for 20 min at 121 °C to ensure sterilization. An orbital agitator (120 rpm) at 30 ± 2 °C was used to inoculate 10 ml of spore suspension of each fungal strain with 50 mg/L of congo red, crystal violet, bromophenol blue, and malachite green. The degradation of dye was evaluated after seven days by measuring the absorbance of each dye at its maximum using a UV–Vis spectrophotometer. Every experiment was repeated three times, and the results were compared to the control. The following formula was used to calculate the percentage of decolorization according to Shah et al. [41]:

$$\text{Percentage of decolorized dye }=\frac{\text{Reading of initial absorbance}-\text{ Reading of final absorbance}}{\text{Reading of initial absorbance}}\times 100$$

#### Determination of growth

The fungal mycelium, the control (untreated), and the treated samples were centrifuged for 10 min at 10,000 × g. The pellets were obtained and then washed three times with sterile distilled water. Then, the fresh weight (FW) was recorded. The biomass was then dried at 60°C until it reached a consistent weight to measure the dry weight (DW).

### Optimization of the decolorizing ability of the most potent fungal isolate

The most potent fungal isolate (*A. flavus*) was used to study the effects of various culture conditions, including incubation periods, pH, temperature, different carbon, nitrogen sources, metals, and static and shaking conditions on the decolorization of congo red, crystal violet, bromophenol blue, and malachite green.

### Effect of different dye concentrations on decolorization percentage (%)

*A. flavus* AUMC8653 was inoculated into Czapek's Dox broth medium after different azo dyes were individually added at various doses (50, 100, 150, and 200 mg/L) as the sole carbon source. After an 8-day incubation period, the highest λ max of each dye was used to calculate the decolorization percentage (%).

### Effect of different incubation periods, pH values, and temperature on decolorization percentage (%)

*A. flavus* AUMC8653 was inoculated into 250 mL conical flasks containing 50 mL of Czapek's Dox medium with the addition of 50 mg/L of each dye. The flasks were placed in an incubator at temperature of 30 ± 2 °C for 2, 4, 6, 8, and 10 days. The percentage of dye decolorization (%) was recorded. Another experiment was carried out using different pH values (5, 6, 7, and 8). After eight days of incubation at 30°C, the percentage of dye decolorization was calculated for each group. Another experiment was conducted by incubating flasks at different temperatures: 20, 25, 30, 35, and 40°C at pH 6. Three independent experiments were performed and the percentage of dye decolorization was determined for each flask.

### Effect of different carbon and nitrogen sources on the dye decolorization

*A. flavus* AUMC8653 was inoculated in 50 ml of Czapek's Dox broth medium containing different carbon sources: glucose, fructose, sucrose, maltose, lactose, or starch with the addition of 50 mg/L of azo dyes, individually. Another experiment was conducted using different nitrogen sources (NaNO_3_, peptone, and yeast extract at 2 g/L and equivalent nitrogen for NH_4_NO_3_, KNO_3_, and urea). All flasks were then incubated at 30 ± 2 °C for 8 days. Each experiment was conducted three times, and the percentage of decolorization was measured.

### Effect of some heavy metals on the decolorization activity

*A. flavus* AUMC8653 was inoculated in 50 ml of Czapek's Dox liquid medium with the addition of 100 ppm concentrations of Mg, Cu, Ca, Ni, and Co, individually. Two sets of experiments were conducted with and without 50 mg/L of azo dyes. Each experiment was repeated three times, and the percentage of decolorization was determined.

#### Effect of static and shaking incubation on dye decolorization

The Czapek Dox liquid medium was inoculated with *A. flavus* AUMC8653 along with the addition of 50 mg/L of azo dyes, individually. The flasks were then incubated at 30 ± 2 °C for 8 days under both static and shaking conditions at an agitation rate of 120 rpm. Afterward, samples were centrifuged at 10,000 rpm for 20 min. The supernatant was utilized to determine the percentage of decolonization.

#### Biochemical and enzyme assay

*A. flavus* AUMC8653 was inoculated in Czapek's Dox liquid medium with the addition of 50 mg/L of azo dyes for 120 h. The resultant pellet (fungal biomass) was utilized to analyze the protein and H_2_O_2_ content, and the resulting supernatant was employed to study extracellular enzymes as a crude enzyme extract.

According to Srinu et al. [42], the laccase activity (EC 1.10.3.2) was measured using guaiacol as the substrate. The absorbance of the sample was measured at 450 nm. In addition, manganese peroxidase (Mnp) (EC 1.11.1.13) was measured using guaiacol as the substrate. MnP activity was determined by measuring the absorbance of the obtained sample at λ465 nm using a UV–Vis spectrophotometer [43]. Lignin peroxidases (LiP, EC 1.11.1.14), the reaction was measured at 310 nm in a UV spectrophotometer [42]. The intracellular protein content was determined according to the method of Lowry et al. [44], and the H_2_O_2_ content of the fungal biomass was assessed using the procedure outlined by Velikova et al. [45].

#### FT-IR

FT-IR analysis investigated the degradation of various azo dyes and assessed the toxicity of degraded compounds through functional group changes. Ethyl acetate was used as a mid-polar solvent for the liquid–liquid extraction of the broken-down metabolites. To eliminate the moisture in the sample, potassium bromide was heated to 105 °C for 30 min. To make it in the ratio of 5:95 (w/w) for analysis, it was further powdered. A total of sixteen scans were conducted in the mid-IR 400–4000 cm^−1^ band to record the results [46].

#### Light microscopy

The mycelia were filtered from Czapek's Dox liquid medium containing azo dyes and photographed using a Microscope.

#### hytotoxicity study

Seeds of maize and common bean were obtained from the Agricultural Research Center in Giza, Egypt. The seeds of maize and common bean were sterilized with 0.1% sodium chlorite for 4 min, followed by five washes with sterile distilled water. Afterward, they were planted in pots filled with 8 kg of air-dried clay loam soil in a completely randomized design (CRD). The pots were split into four groups, each consisting of three replications, and kept at a temperature of 35 °C and a relative humidity between 60–75%. The first group contained only soil irrigated with water, serving as the control. The second group was irrigated with filtrate of *A. flavus*, the third group was irrigated with 50 mg/L azo dyes, and the fourth group was irrigated with azo dyes after biodegradation. The pots were watered every two days. The morphological criteria of the plants were observed 21 days after sowing.

Seed germination percentage, shoot length, root length, fresh and dry weight of seedlings, vigor index I, and vigor index II were measured after 21 days of treatment. Seeds germinated after 7 days were considered as germinated seeds, and germination% was calculated using the following formula [47, 48]:

$$\mathrm {Germination \, \%} = \frac {\mathrm {Number \,of \,seeds \,germinated}} {\mathrm {Total \,number \,of \,seeds}} \times 100$$

Seedling vigor index I and seedling vigor index II were determined using formulas:

$$\mathrm{Vigor \,index \,I }=\mathrm{Germination\, \%}\times \mathrm{ seedling \,length }$$

$$\mathrm{Vigor \,index \,II }=\mathrm{Germination \, \%}\times \mathrm{dry \,biomass }$$

#### Statistical analysis

Each treatment consists of three replicates. The greenhouse experiment was organized using completely randomized designs (CRD). Utilizing SPSS Software version 9, the data were subjected to an ANOVA, and the means of treatments were compared using the Duncan multiple range test (*P* < 0.05).

## Results and discussion

As a result, the need to remediate wastewater contaminated by dyes is becoming essential [49]. It has been challenging for scientists to develop a unified and cost-effective technique for treating dyes in textile effluent [49].

### Screening of the obtained fungal isolates for decolorization of azo dyes

**Fig. 1:**
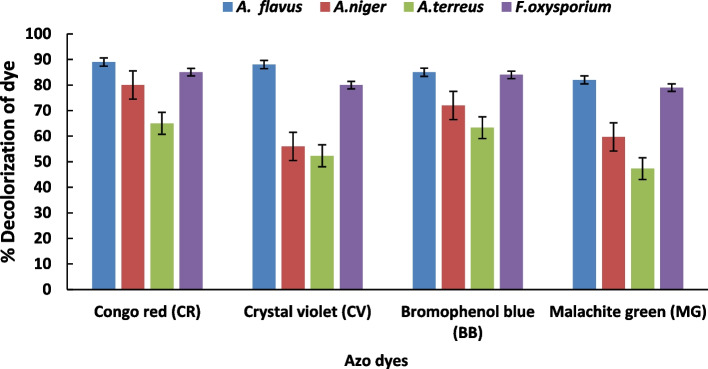
Decolorization of azo dyes (50 mg/L) by different fungal strains. Data represent mean values of three replicates ± show standard error (SE)

The fungal isolates were tested for their ability to decolorize azo dyes at a concentration of 50 mg/L (Fig. 1). The results showed that *A. flavus* exhibited significantly higher decolorization ability followed *F. oxysporium* compared to the other isolates*. A. flavus* showed the greatest decolorization capacity of congo red, crystal violet, bromophenol blue, and malachite green by approximately 89.0%, 88.0%, 85.0%, and 82.0%, respectively. Hefnawy et al. [50] results are in line with this observation. They discovered that *Aspergillus flavus* and *Penicillium canescens* achieved the highest decolorization value of direct blue dye when the initial dye concentration was 0.01%. However, this value decreased at concentrations higher than 0.01%, likely due to dye toxicity.

### Effect of dye concentrations on dry weight of fungal

**Fig. 2:**
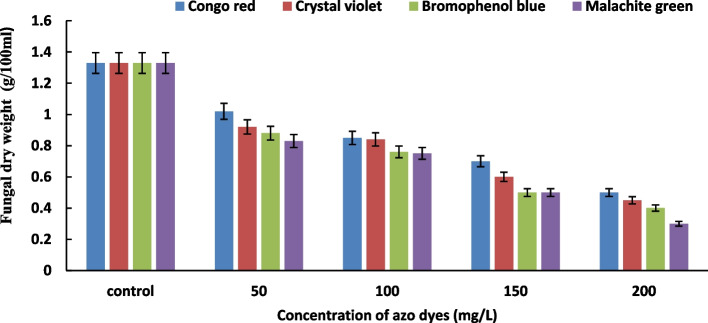
The effect of different concentrations of azo dyes on dry weight of A. flavus. Data represent mean values of three replicates ± show standard error (SE)

The data in Fig. 2 demonstrated that the fungal dry weight (g/100mL) of *A. flavus* AUMC8653 declined above a concentration of 100–200 mg/L and increased at lower concentrations of the dye in the growth medium up to a concentration of 50 mg/L. This indicates that the ability of *A. flavus* to remove dye was diminished as dye concentrations increased. The fungal dry weight increased most noticeably when they were cultivated in medium with 50 mg/L of congo red (1.02g/100mL), crystal violet (0.92g/100mL), bromophenol blue (0.88g/100mL), and malachite green (0.83g/100mL). Congo red consistently resulted in the highest fungal weight among all azo dyes tested. The data presented in Fig. 2 indicates that a dye concentration of 100 mg/L is considered sub-lethal, which negatively affects the dye removal capacity of the four azo dyes. High dye concentrations are toxic to fungal growth and hinder dye decolorization, and there is limited information available about fungal dye decolorization at this concentration. As a result, a dye concentration of 50 mg/L was selected for further investigation in this study. These findings align with Singh and Singh [51] study which found that the supplementation of bromophenol blue and congo red to potato dextrose agar medium individually suppressed the growth of A. flavus compared to their respective controls. Kunjadia et al. [52] found that the increment of crystal violet concentration caused a reduction in the mycelial growth of *Pleurotus ostreatus* MTCC142. Furthermore, increasing the dye concentration decreased the enzymes' ability to degrade azo dyes [53, 54]. Furthermore, 97.41% of the reactive red HE7B dye can be eliminated by *Aspergillus salinarus* strains cultivated on potato dextrose broth medium containing 50 mg/L of the dye [55].

Many studies have shown the toxicity of azo dyes to microorganisms involved in biodegradation. The type, concentration, and dye particle blockage of azo reductase enzyme active sites all affect toxicity [56]. The toxicity of dyes to fungal cells, particularly at elevated concentrations, may result from factors such as high molecular weight, structural complexity, and the presence of inhibitory groups like sulfonic acid [57]. According to Fetyan et al. [58], *Saccharomyces cerevisiae* showed higher decolorizing levels in direct blue71 achieving 100% activity. However, at concentrations exceeding 200 ppm, the rate of decolorization decreased. This decline may be attributed to the potential toxicity of higher concentrations on microbial cells. Furthermore, compared to their respective controls, adding bromophenol blue and congo red to potato dextrose agar media significantly inhibited the growth of *Aspergillus* sp. [50].

### Effect of different incubation periods on dye decolorization percentage

**Fig. 3:**
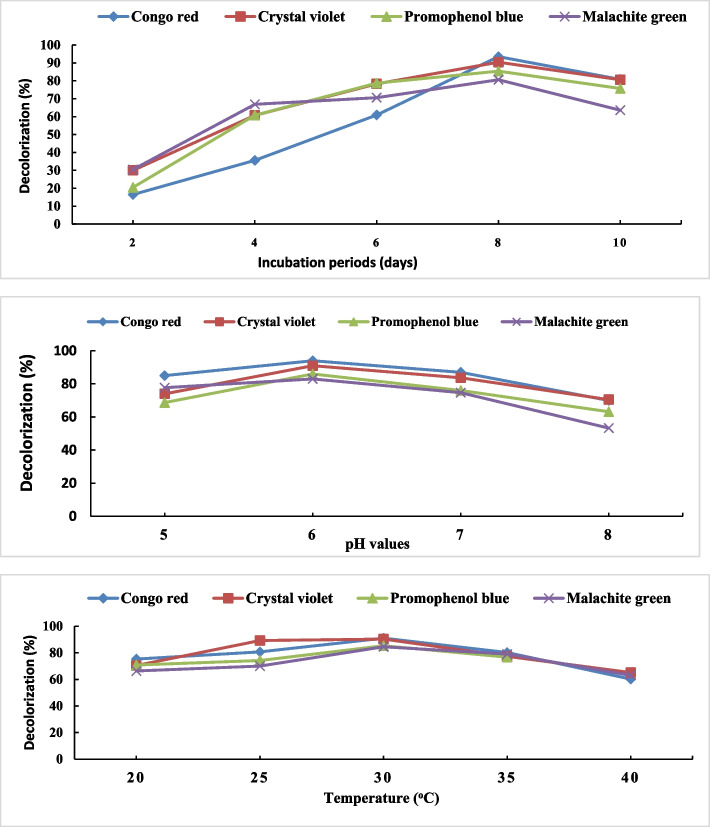
Effect of incubation period, initial medium pH and temperature on decolorization percentage of 50 mg/L of azo dyes by A. flavus

The data presented in Fig. 3 illustrated that extending the incubation period from 2 to 10 days led to a notable increase in the percentage of decolorization across all concentrations of azo dyes used. The highest decolorization percentages were detected for congo red (93.5%), crystal violet (90.4%), bromophenol blue (85.4%), and malachite green (80.6%) dyes during eight days of incubation and 50 mg/L of azo dye. The decolorization of dye through adsorption and breakdown is primarily influenced by the duration of the incubation period [59]. The degree of decolorization significantly decreased both before and after the 8-day incubation period. This decrease in decolorization may be due to the efflux mechanism in fungal cells, which reduces the dye's toxicity. After eight days, there could have been a decline in the decolorization process. The decrease in fungal growth and metabolic activities could have been attributed to enzymatic biodegradation activity, physical binding of dye to fungal biomass, and dye buildup [50].

### Effect of different pH on dye decolorization percentage

The rate of dye decolorization and biomass formation is significantly influenced by pH [60]. The pH level may affect the surface of mycelium, which is composed of biopolymers with various functional groups. Chitin, proteins, polysaccharides, and other compounds present on the mycelium surface contain NH^3+^ and COOH groups. These groups can interact with dye anions through electrostatic interactions [60]. Our results found that *A. flavus* is most effective at decolorizing azo dyes at pH 6 (Fig. 3). This suggests that the acidic pH range is ideal for *A. flavus* to decolorize these dyes. According to our findings, *A. flavus* AUMC8653 was able to decolorize dyes as follows: 94.0% of congo red, 91.0% of crystal violet, and 86.0% of malachite green at pH 6.0. The results align with those reported by Hefnawy et al. [50] who found that *A. flavus* can thrive and decolorize within a broad pH range of 3 to 8, with the optimum pH being 4. This indicates that the strain can be valuable for treating wastewater and for various industrial uses. Additionally, these findings suggest that acidic pH levels may affect the stability of the enzymes [59]. The decolorization efficiency of *A. flavus* A5p1 declines significantly as pH increases [61], due to the interaction of positively charged cell surfaces with negatively charged dye anions, as demonstrated in the instance of congo red and coir pith carbon [62]. Dyes tend to decolorize at higher concentrations in acidic environments, which facilitates their removal through enzymatic activity or adsorption onto the fungal cell wall, according to Namdhari et al. [57]. It is well known that [H +] ions regulate dye degradation, which is influenced by the pH of the medium.

### Effect of different temperatures on dye decolorization percentage

The impact of incubation temperature on the decolorization of 50 mg/L of azo dyes was investigated throughout a temperature range of 20–40°C. According to the results, 30°C was the most effective temperature for decolorizing congo red (91.0%), crystal violet (90.3%), bromophenol blue (85.3%), and malachite green (84.5%) dyes. At higher temperatures (40°C), the decolorization % was decreased (Fig. 3). This decrease may be due to the loss of cell viability [50] or to the denaturation of ligninolytic enzymes. This means that the rate of color removal increases with temperature [50, 63]. The azo dye reactive violet 1 decolorization efficacy of *Ganoderma cupreum* AG-1 was higher at lower temperatures (25–35°C) [64]. According to the study of Ameen et al. [64] who found that the optimum temperature range for *Aspergillus* strains to decolorize dyes was 30–35°C. The optimum temperature for each organism promotes growth and metabolic activities. Furthermore, Singh and Dwivedi [65] found the maximum decolorization of congo red (92.60%) with *A. flavus* treatment at pH 6.0, 30°C, 120 rpm stirring speed, and 120-h duration. During the adsorption or decolorization process, the ideal temperature critically influences the kinetic energy and surface energy of dye molecules. It is also necessary for the release of enzymes during degradation that break down dye [66, 67].

### Effect of different carbon sources on dye decolorization

**Fig. 4:**
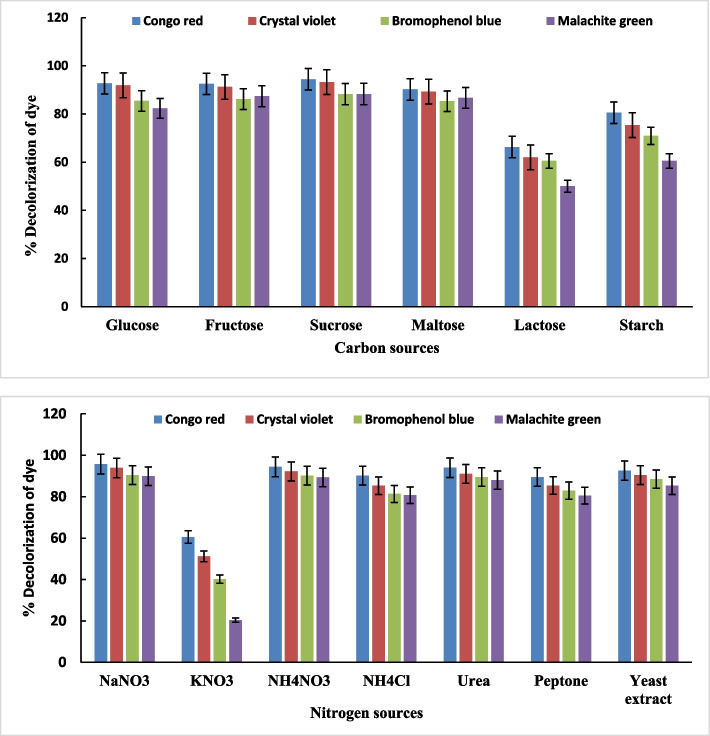
Effect of different carbon and nitrogen sources on decolonization of 50 mg/L of azo dyes by A. flavus. Data represent mean values of three replicates ± show standard error (SE)

Fungi need nutrient supplements like carbon and nitrogen to grow and to break down contaminants. Glucose, fructose, and sucrose have been extensively studied as carbon sources for fungal growth; while starch and xylan also appear to be important carbon sources [68]. The data in Fig. 4 presented that *A. flavus* AUMC8653 could effectively use certain carbon sources such as fructose, sucrose, glucose, and maltose to achieve high decolorization percentages. However, the growth of *A. flavus* AUMC8653 was decreased in the presence of starch and lactose, resulting in lower decolorization values. Sucrose was identified as the best carbon source for *A. flavus* AUMC8653, leading to the highest decolorization percentages for congo red (94.4%), crystal violet (93.2%), bromophenol blue (88.2%), and malachite green (88.3%) dyes. Similar results were observed in a study by Hefnawy et al. [50] when direct blue dye decolorization was evaluated after *A. flavus* and *P. canescens* were grown in various carbon sources, such as glucose, sucrose, fructose, or maltose. These factors are crucial for the efficient breakdown of dyes [69, 70]. The ability of microbes to break down and decolorize azo dye depends on the types and availability of carbon–nitrogen sources, which serve as electron donors during the decolorization process [24].

### Effect of different nitrogen sources on dye decolorization

The highest decolorization percentage was achieved when NaNO_3_, NH_4_NO_3_, urea, and yeast extract were supplemented with the growth medium of *A. flavus* (Fig. 4). It was also noted that the lowest decolorization percentage of various azo dyes was obtained in the presence of KNO_3_ in the growth medium. On the other hand, NaNO_3_ was identified as the most effective carbon source utilized by *A. flavus* AUMC8653, leading to the highest decolorization percentages for congo red (95.7%), crystal violet (93.9%), bromophenol blue (90.4%), and malachite green (89%). Similar results were published by Hefnawy et al. [50], who noted that *A. flavus* and *P. canescens* displayed substantial growth and decolorization percentages of direct blue dye when using all nitrogen sources except for KNO_3_. The ideal concentration of nitrogen sources was 0.5% of ammonium sulfate and 0.2% of sodium nitrate for the degradation of azo dyes by *Trichoderma virens, Phlebiopsis cf. ravenelii, Talaromyces stipitatus*, and *A. niger* [71].

### Effect of some heavy metals on the dye decolorization

**Fig. 5:**
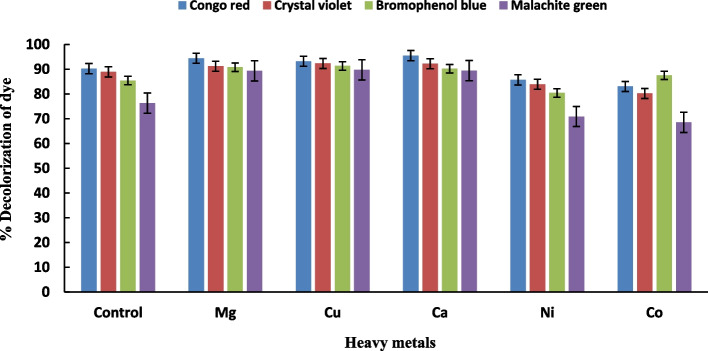
Effect of different heavy metals (100 ppm) on decolonization of azo dyes by A. flavus. Data represent mean values of three replicates ± show standard error (SE)

Metal ions are considered to be potential laccase inhibitors because these ions bind to the enzymes and de/stabilize the protein and they change the enzyme activity [50, 65]. The results in Fig. 5 revealed that Mg^+2^, Ca^+2^, and Cu^+2^ substantially improved the decolorization of azo dyes compared to the control. In contrast, the presence of Ni^+2^ and Co^+2^ in the growth medium resulted in a decrease in the percentage of color reduction. Ca^+2^ proved to be the most effective metal utilized by *A. flavus* AUMC8653, resulting in the highest decolorization percentages for congo red (95.5%), crystal violet (92.2%), bromophenol blue (90.2%), and malachite green (89.4%) compared to the control. However, it is well known that heavy metals inhibit several enzymes from the primary and secondary metabolic pathways. However, the percentage of color reduction was lower in the presence of Ni and Co in the growth medium. The decolorization activity of *A. flavus* was enhanced by Ca, Ni, or Co, but inhibited by Cu, while, the decolorization activity of *P. canescens* was significantly decreased by these metals, especially Cu [50]. Singh and Dwivedi [65] found that *A. terreus* GS28 isolate showed the highest decolorization direct blue-1 of 94.67% with manganese, 85.56%-83.88% with copper and zinc, and the lowest decolorization of 70.26% with iron. Manganese induced the activity of manganese peroxidase in isolate *A. terreus*, which enhanced the degradation of direct blue-1 dye. Similarly, copper enhanced laccase activity, which also contributed to the degradation of the direct blue-1 dye [65].

### Effect of static and shaking incubation on dye decolorization

The dyes (congo red, crystal violet, bromophenol blue, and malachite green) were decolorized by *A. flavus* during static and shaking incubations. There are notable variations in the decolorization of different azo dyes by *A. flavus*. However, the static culture generally performed better than the shaking condition in terms of both dye removal and fungal growth (Table 1). The treatment with congo red resulted in higher fresh weight (11.4 g), dry weight of fungal (2.3 g), and decolorization percentage (96.8%) under static culture. The decolorization of dye in stationary cultures was caused by the absorption of the dyes on the fungal biomass, which was not detected in agitated cultures. Bettin et al. [72] used laccases from *Pleurotus sajorcaju* PS-2001 to study the decolorization of 22 different colors. They found that under the same pH (3.2) and temperature (30–30 °C), the decolorization percentages were much lower during agitation (100 min^−1^) compared to static conditions.

**Table 1:** Growth of *A. flavus* and dye decolorization in static and shaking culture (fresh weight, dry weight, and dye decolorization)

	Static culture			Shaking culture		
Treatment	Fresh weight (g)	Dry weight (g)	% Decolorization of dye	Fresh weight (g)	Dry weight (g)	% Decolorization of dye
Control	11.8 ± 0.2^a^	2.3 ± 0.01^a^	0	10.12 ± 0.3^a^	1.43 ± 0.01^a^	0
Congo red	11.4 ± 0.2^a^	2.0 ± 0.02^a^	96.8 ± 1.2^a^	9.49 ± 0.2^b^	1.20 ± 0.01^b^	94.4 ± 1.5^a^
Crystal violet	10.9 ± 0.1^b^	2.1 ± 0.01^b^	94.2 ± 1.0^b^	9.21 ± 0.2^b^	1.02 ± 0.01^c^	92.3 ± 1.3^b^
Bromophenol blue	7.7 ± 0.1^c^	1.2 ± 0.01^c^	74.4 ± 0.8^c^	7.07 ± 0.1^c^	0.89 ± 0.01^d^	89.4 ± 1.2^c^
Malachite green	7.0 ± 0.1^d^	0.8 ± 0.01^d^	70.5 ± 0.5^d^	5.38 ± 0.1^d^	0.89 ± 0.01^d^	89.4 ± 1.0^c^

Data represent mean values of three replicates ± show standard error (SE). Means with different letters in the same column were significantly differ at *p* ≤ 0.05 according to Duncan’s Multiple Range Test

#### Effect of azo dyes on enzyme activities

The release of several oxidoreductases, such as laccase, peroxidase, and azoreductase, enables microorganisms to remove dyes using enzymes, as previously documented [73, 74]. Oxidoreductases efficiently break down dyes and produce more environmentally friendly products. The cell walls of fungi contain various components, including heteropolysaccharides such as chitin, chitosan, and glucan, as well as lipids and phospholipids [75, 76]. These components serve as binding sites for functional groups like carboxyl, hydroxyl, and phosphoryl groups, which facilitate the biosorption process [77]. Additionally, filamentous fungi are a powerful source of enzymes capable of converting a wide range of colors. Fungal enzymes that are known to contribute to the breakdown of dyes include laccases, lignin peroxidases, and manganese peroxidases [78].

The results in Table 2 show a significant increase in laccase and manganese peroxidase in the supernatant of *A. flavus* AUMC8653 supplemented with different azo dyes compared to the supernatant of *A. flavus* without azo dyes. The most pronounced increases of MnP activity were detected in the supernatant of A. flavus AUMC8653 supplemented with congo red (3.3μm/L), crystal violet (2.9μm/L), bromophenol blue (2.5μm/L), and malachite green (2.3μm/L), respectively compared to the control after 7 days. According to Ning et al. [61], similar findings demonstrated a 3.2-fold increase in MnP activity with the presence of Direct Blue 86. It was suggested that the dye may act as an activator, enhancing the production of important enzymes by *A. flavus* A5p1. Moreover, manganese peroxidase (MnP) is regarded as an essential enzyme that plays a significant role during the elimination process [61]. The decolorization process involves various oxidoreductase enzymes, which transform the original dye molecule into a less harmful substance [77]. MnP belongs to the lignolytic peroxidase family and can oxidize phenolic compounds by converting Mn^2+^ to Mn^3+^ and mineralize azo dyes through redox reactions [65]. The Mn^3+^ compounds are active oxidants, which are typically stabilized by chelating organic acids such as oxalic acid [65].

**Table 2:** Enzymatic and biochemical parameters under 50 mg/L of azo dyes by *A. flavus*

Treatment	Laccase (μm/L)	Manganese peroxidase (μm/L)	Lignin peroxidase (μm/L)	Protein (mg/g FW)	H_2_O_2_ content (nM/ g FW)
Control	0.40 ± 0.01^e^	1.1 ± 0.01^e^	1.9 ± 0.02^d^	0.20 ± 0.01^a^	0.20 ± 0.01^e^
Congo red	0.80 ± 0.02^a^	3.3 ± 0.03^a^	4.2 ± 0.05^a^	0.18 ± 0.01^b^	1.01 ± 0.08^d^
Crystal violet	0.75 ± 0.02^b^	2.9 ± 0.03^b^	3.3 ± 0.05^b^	0.16 ± 0.01^c^	2.3 ± 0.1^c^
Bromophenol blue	0.60 ± 0.02^c^	2.5 ± 0.02^c^	3.0 ± 0.04^c^	0.14 ± 0.01^d^	2.9 ± 0.1^b^
Malachite green	0.55 ± 0.01^d^	2.3 ± 0.01^d^	2.9 ± 0.02^c^	0.13 ± 0.01^d^	3.8 ± 0.1^a^

Data represent mean values of three replicates ± show standard error (SE). Means with different letters in the same column were significantly differ at p ≤ 0.05 according to Duncan’s Multiple Range Test

Our results showed that the higher concentrations of laccase enzyme were detected in the supernatant of *A. flavus* AUMC8653 supplemented with congo red (0.80μm/L), crystal violet (0.75μm/L), bromophenol blue (0.60μm/L), and malachite green (0.55 μm/L), respectively compared to the control after 7 days (Table 2). The process of dye degradation by fungi is straightforward, as it involves the oxidation of complex pollutants, such as azo dyes, into simpler compounds. This occurs due to the production of extracellular enzymes, including laccase [16]. Laccases are phenolic oxidases that contain copper ions, which contribute to the generation of non-toxic phenolic compounds during biodegradation. This process operates through a non-specific free radical mechanism. In the degradation of azo dyes, the primary steps include the breakdown of the N–N bond, along with deamination, oxidation, desulfonation, and dihydroxylation [16, 79].

Our results showed that the higher concentrations of lignin peroxidase (LiP) enzyme were detected in the supernatant of *A. flavus* AUMC8653 supplemented with congo red (4.2μm/L), crystal violet (3.3μm/L), bromophenol blue (3.0μm/L), and malachite green (2.9μm/L), respectively compared to the control after 7 days (Table 2). Lignin peroxidases decompose dyes by oxidizing the phenolic group attached to the azo bond-bearing carbon, which generates a radical group. This phenolic carbon is then attacked by water, leading to the formation of phenyldiazene. Subsequently, phenyldiazene can be oxidized through a one-electron process, resulting in the production of nitrogen gas (N_2_). LiP increased degradation capacity in the presence of H_2_O_2_ [76].

The supernatant of *A. flavus* AUMC8653 was supplemented with different azo dyes and showed a significant increase in hydrogen peroxide compared to the supernatant of *A. flavus* without azo dyes. In addition, the maximum H_2_O_2_ content (3.8nM/g FW) was observed at 50 mg/L of malachite green treated with *A. flavus* AUMC8653. The minimum H_2_O_2_ content (1.01nM/g FW) was observed at 50 mg/L of congo red treated with *A. flavus* AUMC8653. Comparable outcomes were stated by Singh and Dwivedi [65], who observed that the highest H_2_O_2_ content was seen at 100 mg/L of direct blue-1 dye-treated *A. terreus* fungal biomass, followed by 500 and 1000 mg/L of direct blue-1 dye. Manganese peroxidase is part of a similar family, and in this study, the slow release of H_2_O_2_ boosted the activity of manganese peroxidase. When there is a lack of nutrients, mycelia produce H_2_O_2_, which activates the lignolytic enzymes for complete substrate degradation [80]. Enzymatic activity has a role in the decolorization of congo red and is indirectly linked to the generation of H_2_O_2_ [24]. H_2_O_2_ has a special function in the radical-generating process that breaks down lignocellulose [65]. Although a significant amount of H_2_O_2_ is necessary for peroxidase activity as a co-factor, it can occasionally also impede enzymatic activities [74]. Some fungal strains, such as *A. fumigatus* and *A. flavus*, induce degradation and produce water as a byproduct. Similarly, peroxidases in fungi utilize hydrogen peroxide (H_2_O2) as an oxidizing agent [16].

The protein content of A. flavus decreased significantly in congo red (0.18 mg/g), crystal violet (0.16 mg/g), bromophenol blue (0.14 mg/g), and malachite green (0.13 mg/g) as compared to the control (Table 2). Similar results were obtained by Singh and Dwivedi [65] who reported that the protein content of *A. terreus* showed increasing trends with 100, 500, and 1000 mg/L of direct blue-1 dye, but was lower than the control. This could be due to the generation of more stress at increasing concentrations of direct blue-1 dye, while the control showed the maximum protein content without the stress of the dye.

#### FT-IR

FT-IR studies showed that this was mostly caused by laccase-mediated enzymatic breakdown, which produced less hazardous metabolites. The degradation process involved the conversion of toxic malachite green to benzaldehyde via Michler's ketone pathway [79]. Additionally, another study reported the biodegradation of malachite green using *A. flavus*, which successfully degraded malachite green completely within 8 days by synthesizing laccases and manganese peroxidase [81].

**Fig. 6:**
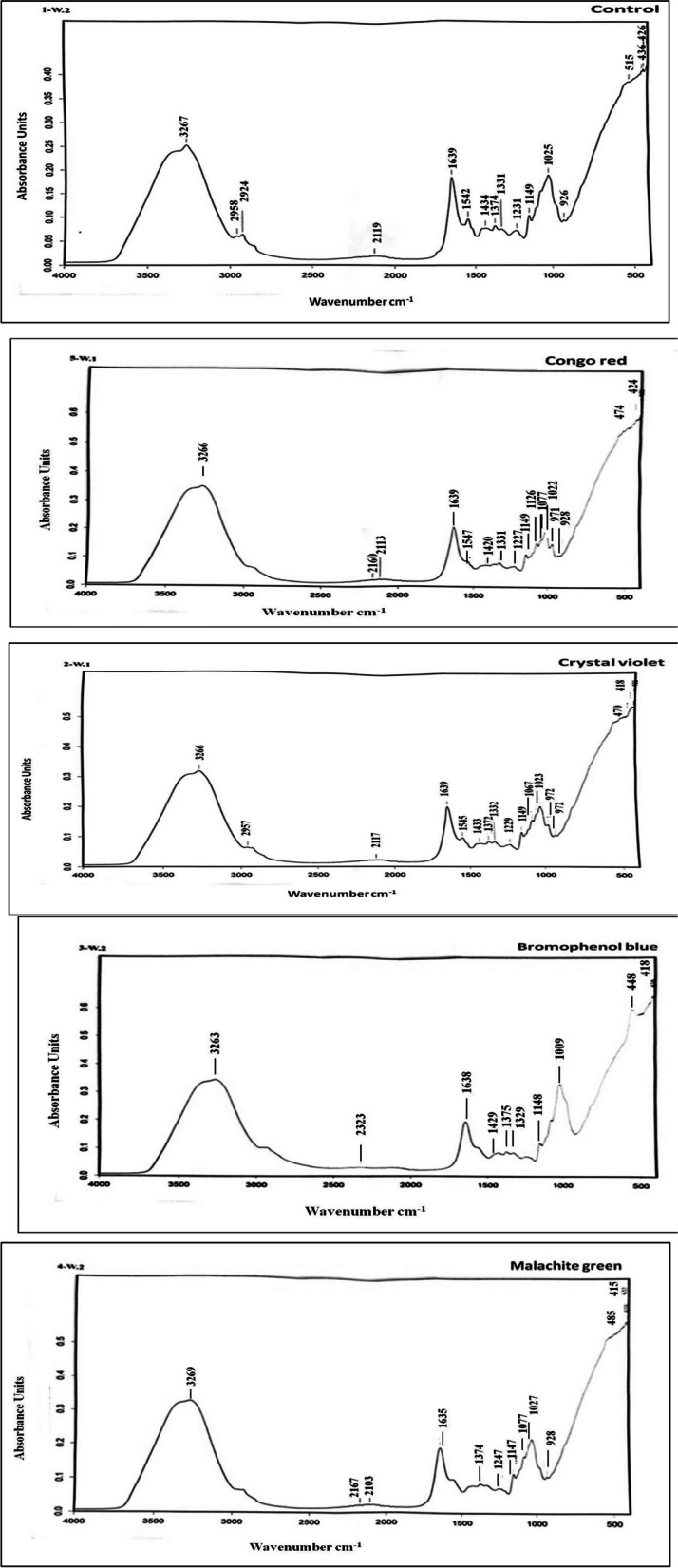
Exemplary FT-IR band positions (cm-1) of A. flavus before and after biodegradation of azo dyes

The functional groups and chemical bonds in *A. flavus* were analyzed before and after dye biodegradation using FT-IR spectroscopy. Based on Fig. 6a, the FT-IR spectrum of *A. flavus* before the azo dyes removal displays several major intense bands, around 3267, 2958, 2924, 2219, 1639, 1542, 1434, 1374, 1331, 1231, 1149, 1025, 926, 915, 436 and 426 cm^−1^. The peak at 3267 cm^−1^ indicated that there was a strong absorption band due to the symmetric stretching vibration of O–H and NH_2_ groups, while, the peak at 1542 cm^−1^ indicated the presence of –NH in *A. flavus*. The C–H asymmetric stretching peak is located at 2924 cm^−1^, the C = O stretching and N–H deformation peak is located at 1639 cm^−1^ (in the amide II area), the carboxyl group is located at 1542 cm^−1^, and the phosphate group is located at 1231 cm^−1^. The peaks at 1148 cm^−1^ correspond to groups containing phosphate and sulfide. The polysaccharide groups C–C, C = C, C–O–C, and C–O–P are represented by the peaks at 1025 cm^−1^.

Referring to Table 3 and Fig. 6 b-e, following the biodegradation of azo dyes, certain peaks exhibited minimal variation; some peaks appeared, while other peaks disappeared. This behavior was explained as a result of the azo dyes aligning with an active functional group, which changed the vibration frequency. Furthermore, positively charged azo dyes can bind to the cell surface through the electrostatic force produced by negatively charged functional groups (such as hydroxyl, amino, carboxyl, phosphate, nitro, and halide groups).

**Table 3:** Exemplary FT-IR band positions (cm^−1^) of *A. flavus* before and after biodegradation of azo dyes

Control	Congo red (CR)	Crystal violet (CV)	Bromophenol blue (BB)	Malachite green (MG)	Suggested assignment
3267	3266	3266	3263	3269	O–H and N–H stretching
2958	-	2957	-	-	C-H alkyl and aldehyde group
2924	-	-		-	C–H asymmetric
-	-	-	2323	-	ketenes, O = C = O
-	2160	-	-	2167	S-C≡N stretching thiocyanate
2119	2113	2117	-	2103	N = N = N stretching azide
1639	1639	1639	1638	1635	C = O stretching and N–H deformation (amide II region)
1542	1547	1545	-	-	Carboxyl group
1434	1420	1433	1429	-	C–N stretching and N–H bending of amide III
1374	-	1372	1376	1374	Aliphatic amines (CH_3_)
1331	1331	1332	1329	-	O–H bending alcohol
1231	1227	1229	-	1247	Phosphate group
1149	1149	1149	1148	1147	Phosphate and sulfide groups
-	1126	-	-	-	Aromatic compounds
-	1077	1076	1009	1077	C–N stretching vibration of amine groups
1025	1022	1023	-	1027	C–C, C = C, C–O–C, and C–O–P groups of polysaccharides
-	971	972	-	-	C = C bending alkene
926	928	927	-	928	Si–O stretching
515	-	-	-	-	
-	474	470		485	phosphate's (PO_4_^3−^)
436	-	-	448	-	
426	424	418	418	415	O-Si–O

‘‘–’’ denotes the peak disappeared in dye-treated fungal biomass

Following dye biodegradation, the FT-IR spectra are shown in Fig. 6b. The FT-IR peaks shift as a result of interactions between congo red molecules and *A. flavus* active surface during adsorption. FT-IR spectrum shows multiple strong bands at around 3266, 2113, 1639, 1547, 1420, 1331, 1227, 1149, 1022, 928, and 424 cm^−1^. Along with the elimination of several bands around 2985, 2924, 1374, 915, and 436 cm^−1^, the FT-IR spectra also showed the emergence of additional bands around 2160, 1126, 1077, 971, and 474 cm^−1^. Similar results were published by Kaushik and Seth [66], who demonstrated that the degradation of congo red (CR) and malachite green (MG) was validated by comparing the FT-IR spectra of the control dye sample and the samples extracted following decolorization by *A. flavus* strain GKRS09. Dye degradation was indicated by the FT-IR study's decrease in peak spectra, which shifted between the treated and control dyes.

The FT-IR spectra following dye biodegradation are shown in Fig. 6c. The FT-IR peaks shift as a result of interactions between crystal violet molecules and *A. flavus* active surface upon adsorption. Following the elimination of the crystal violet, *A. flavus* FT-IR spectrum shows multiple prominent bands at approximately 3266, 2957, 2117, 1639, 1545, 1433, 1372, 1332, 1229, 1149, 1023, 927, and 418 cm^−1^. Additionally, the FT-IR spectra show the emergence of new bands at around 1076 and 470 cm^−1^ as well as the loss of existing bands at approximately 2924 and 436 cm^−1^.

FT-IR spectra following dye biodegradation were displayed in Fig. 6d. The interaction between bromophenol blue molecules and *A. flavus* active surface during adsorption causes the FT-IR peaks to shift. *A. flavus* FT-IR spectrum shows multiple prominent bands at approximately 3263, 1638, 1429, 1376, 1329, 1148, 448, and 418 cm^−1^ following the elimination of bromophenol blue. Additionally, the FT-IR spectra shows the emergence of new bands at approximately 2323 and 1009 cm^−1^ as well as the elimination of existing bands at around 2958, 2924, 2119, 1542, 1231, 1025, 926, and 915 cm^−1^.

The FT-IR spectra following dye biodegradation are shown in Fig. 6e. The shift in FT-IR peaks is caused by interactions between malachite green molecules and *A. flavus* active surface following adsorption. Following the elimination of malachite green, the FT-IR spectrum of *A. flavus* shows multiple strong bands around 3263, 2103, 1635, 1374, 1247, 1147, 1027, 928, and 413cm^−1^. The FT-IR spectrum also represents the appearance of new bands around 2167, 1077, 485, and 413 cm^−1^ and the disappearance of other bands around 2958, 2924, 1542, 1434, 1331, 915, and 436 cm^−1^. The metabolites produced when *Lasiodiplodia* sp. broke down malachite green dye were found to have fewer harmful components compared to the control group, according to FT-IR analysis of those metabolites. The analysis also revealed that malachite green dye is transformed into alkanes, carboxylic acids, and aromatic amines. This conversion could be a potential mechanism for *Lasiodiplodia* sp. degradation of malachite green dye [60]. The C = O and N–H stretch cause the formation of amines, amides, and carboxylic acids. The breakdown of MG into less hazardous metabolites is confirmed by the presence of dimer OH [82].

The emergence of new peaks and the disappearance of prominent IR peaks may indicate the mineralization of the parent compound (dye molecules) [80]. The C-N bond breaking and ammonium ion deformation could be indicated by the peaks disappearing [73]. Fungal cell walls in microorganisms contain a variety of charged functional groups, or active sites, including phosphate, hydroxyl, and carboxyl. Fungi release heteropolysaccharides, which are crucial to the adsorption process of indigo decolorization [83, 84]. Since* A. flavus* biomass showed increased peaks impacting the parent chemical of congo red, it proved to be more effective for the surface sorption of congo red compared to* A. terreus* biomass [24].

The ability of fungal mycelia to biosorb is closely linked to their surface area and the functional groups present on their surface [65]. The effectiveness of dye biosorption relies on the hetero-polysaccharide and lipid content of the cell wall. These components possess varied charged functional groups that establish a strong attractive interaction between azo dyes and the cell wall [65]. However, positively charged and negatively charged groups are important to attract both basic and acidic dyes through electrostatic attraction which is the basis of the biosorption mechanism of dye removal [65]. The biotransformation of azo dyes into several metabolites is indicated by the disappearance of the principal IR peak in the FT-IR spectra and the appearance of a new peak [85]. Azo dyes have positively charged nitrogen atoms that interact with negatively charged groups, such as carboxylates and phosphates on the cell wall. Chemical bonds, either covalent or hydrogen, can form a link between azo dyes and the cell wall [86].

#### Light microscopy

**Fig. 7:**
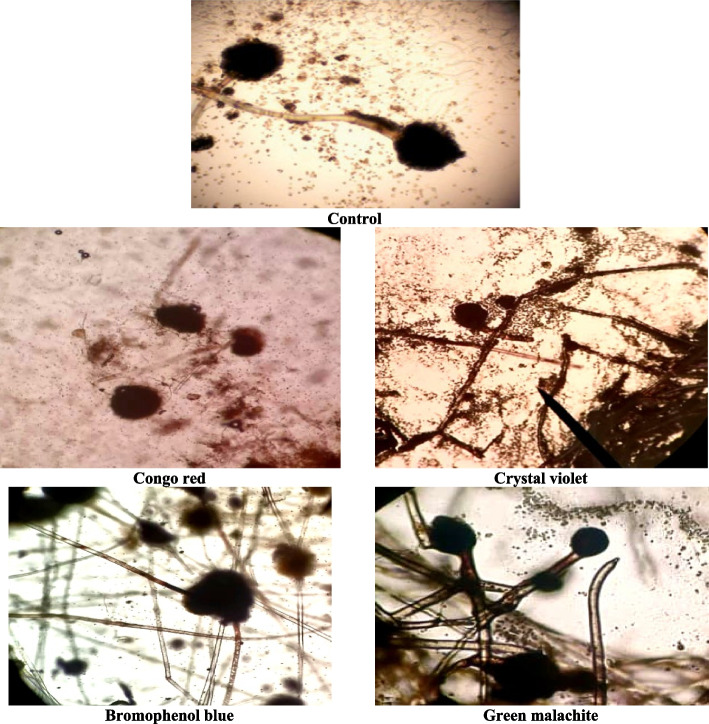
Uptake the dyes inside the fungal biomass of A. flavus after 7 days of incubation with different azo dyes

The findings are supported by the visual observation that the fungal cells have lost their original color. As a result, fungus mycelia are examined under a microscope to determine the intracellular fungal biomass, as depicted in Fig. 7. The findings show that *A. flavus* may accumulate the dyes within the fungal biomass without forming biotransformations, and this process would be the primary mechanism responsible for decolorization. Recent research discovered that *A. flavus* can accumulate dyes in its biomass without altering them, which is the primary mechanism for color removal. The dyes are only adsorbed on the surface of live cells, and fungal enzymes break down the dyes to create new chemicals. When cell pellets are brightly colored, it indicates dye adsorption, while the original color of the dye is preserved when biodegradation occurs [87].

#### Phytotoxicity test

The untreated wastewater from dye factories is found in water bodies that are used for irrigation. This poses serious environmental concerns due to its impact on the health of plants and ecosystems. Water used for irrigation directly and indirectly affects soil fertility, when the wastewater biodegrades, it produces numerous degradation products. It is important to study the toxic effects of these breakdown products on plants [59]. The data in Tables 4 and 5 illustrated the effects of biodegradable azo dyes before and after treatment with *A. flavus* AUMC8653 on seed germination and growth of maize and common bean plants. It was found that the percentage of germination, shoot length, root length, seedling length, fresh weight of seedlings, dry weight of seedlings, vigor index I, and vigor index II increased significantly in plants treated with fungal metabolites. The increase in the germination percentage of both tested plants could be attributed to the less toxic nature of the dye residues and produced metabolites [88]. In addition, all morphological parameters showed a significant reduction when the plants were irrigated with azo dyes compared to control plants. On the other hand, irrigation of plants with azo dyes after biodegradation significantly increased all morphological parameters of maize and common bean plants compared to azo dyes before biodegradation. The inhibition of germination can be attributed to reduced water intake by seeds in the presence of high concentrations of wastewater. This, in turn, affects energy-forming compounds, total solids, and heavy metals [59].

**Table 4:** Phytotoxicity test of degraded products of azo dyes to plant seeds of *Z. mays* after 21 days

Treatment	Germination %	Shoot length (cm)	Root length (cm)	Seedling length (cm)	Fresh weight of seedlings (g)	Dry weight of seedlings (g)	Seedling Vigor I	Seedling Vigor II
Control	100 ± 2.0^a^	25.35 ± 0.3^a^	17.33 ± 0.1^a^	42.68 ± 1.2^b^	1.40 ± 0.01^b^	0.51 ± 0.01^ab^	4268 ± 20.^2a^	51 ± 1.0^a^
*A. flavus*	100 ± 3.0^a^	27.70 ± 0.4^b^	18.55 ± 0.2^a^	46.25 ± 1.5^a^	1.55 ± 0.02^a^	0.57 ± 0.01^a^	4625 ± 22.5^a^	57 ± 1.1^a^
Congo red	35 ± 0.5^c^	12.00 ± 0.1^e^	6.10 ± 0.1^d^	18.10 ± 0.5^e^	0.43 ± 0.01^f^	0.12 ± 0.01^d^	633.5 ± 12.3^e^	5.16 ± 0.2^e^
Degraded congo red	94 ± 1.0^ab^	23.85 ± 0.2^c^	14.92 ± 0.1^b^	38.77 ± 1.6^c^	1.35 ± 0.05^c^	0.47 ± 0.01^b^	3644.38 ± 17.0^b^	44.18 ± 0.9^b^
Crystal violet	30 ± 0.3^ cd^	11.00 ± 0.1^e^	5.50 ± 0.05^d^	16.50 ± 0.6^e^	0.40 ± 0.01^ g^	0.10 ± 0.01^e^	495 ± 12.0^e^	3.0 ± 0.2^e^
Degraded crystal violet	91 ± 0.6^b^	22.70 ± 0.1^ cd^	12.25 ± 0.1^c^	34.95 ± 1.0^d^	1.30 ± 0.02^d^	0.42 ± 0.01^b^	3180.45 ± 25.0^c^	38.22 ± 1.0^c^
Bromophenol blue	27 ± 0.2^d^	10.50 ± 0.1^e^	5.10 ± 0.05^de^	15.60 ± 0.4^e^	0.37 ± 0.01^ h^	0.09 ± 0.01^ef^	421.2 ± 10.0^e^	2.43 ± 0.2^e^
Degraded bromophenol blue	88 ± 1.2^b^	20.33 ± 0.3^d^	13.77 ± 0.2^bc^	34.10 ± 0.9^d^	1.20 ± 0.01^de^	0.40 ± 0.01^bc^	3000.8 ± 12.5^ cd^	35.2 ± 1.3^ cd^
Malachite green	25 ± 0.6^d^	7.22 ± 0.06^f^	4.40 ± 0.02^e^	11.62 ± 0.4^f^	0.33 ± 0.01^j^	0.07 ± 0.01^f^	290.5 ± 10.0^e^	1.75 ± 0.01^e^
Degraded malachite green	85 ± 1.0^b^	20.22 ± 0.4^d^	11.40 ± 0.2^c^	31.62 ± 1.0^d^	1.10 ± 0.01^e^	0.36 ± 0.01^c^	2687.7 ± 24.0^d^	30.6 ± 0.4^d^

Data represent mean values of three replicates ± show standard error (SE). Means with different letters in the same column were significantly differ at p ≤ 0.05 according to Duncan’s Multiple Range Test

**Table 5:** Phytotoxicity test of degraded products of azo dyes to plant seeds of *Phaseolus vulgaris* after 21 days

Treatment	Germination %	Shoot length (cm)	Root length (cm)	Seedling length (cm)	Fresh weight of seedlings (g)	Dry weight of seedlings (g)	Seedling Vigor I	Seedling Vigor II
Control	100 ± 1.9^a^	20.4 ± 0.1^a^	6.5 ± 0.1^ab^	26.9 ± 0.5^b^	1.11 ± 0.01^b^	0.40 ± 0.01^b^	2690^ab^	40.0^a^
*A. flavus*	95 ± 2.1^a^	22.0 ± 0.2^a^	7.0 ± 0.2^a^	29.0 ± 0.6^a^	1.30 ± 0.02^a^	0.45 ± 0.01^a^	2755^a^	42.8^a^
Congo red	40 ± 0.9^c^	10.0 ± 0.1^c^	3.0 ± 0.1^c^	13.0 ± 0.5^d^	0.40 ± 0.01^d^	0.12 ± 0.01^c^	520^d^	4.8^c^
Degraded congo red	96 ± 1.8^a^	21.5 ± 0.3^a^	6.8 ± 0.1^a^	28.3 ± 0.2^ab^	1.27 ± 0.02^a^	0.42 ± 0.01^ab^	2717^a^	40.3^a^
Crystal violet	35 ± 0.6^ cd^	9.3 ± 0.1^c^	2.8 ± 0.1^c^	12.1 ± 0.1^d^	0.38 ± 0.01^d^	0.10 ± 0.01^c^	424^d^	3.5^c^
Degraded crystal violet	92 ± 1.2^b^	20.1 ± 0.2^ab^	6.6 ± 0.1^a^	26.7 ± 0.5^bc^	1.12 ± 0.02^b^	0.40 ± 0.01^b^	2456^b^	36.8^ab^
Bromophenol blue	32 ± 0.8^d^	8.5 ± 0.1^ cd^	2.5 ± 0.1^ cd^	11.0 ± 0.1^de^	0.32 ± 0.01^d^	0.08 ± 0.01^ cd^	352^de^	2.56^c^
Degraded bromophenol blue	90 ± 1.5^b^	19.9 ± 0.2^b^	6.0 ± 0.1^b^	25.9 ± 0.4^b^	1.05 ± 0.01^bc^	0.38 ± 0.01^b^	2331^bc^	34.2^b^
Malachite green	33 ± 0.4^d^	8.0 ± 0.1^d^	2.2 ± 0.01^d^	10.2 ± 0.2^e^	0.30 ± 0.01^d^	0.07 ± 0.01^d^	337^e^	2.31^c^
Degraded malachite green	88 ± 0.9^b^	18.5 ± 0.1^b^	5.7 ± 0.1^b^	24.2 ± 0.5^c^	1.02 ± 0.01^c^	0.36 ± 0.01^b^	2130^c^	31.7^b^

Data represent mean values of three replicates ± show standard error (SE). Means with different letters in the same column were significantly differ at p ≤ 0.05 according to Duncan’s Multiple Range Test

According to Singh and Dwivedi [65], the germination index of *S. leucopersicum* and *T. aestivum* seeds was approximately 36% and 40%, respectively, when exposed to Since *A. flavus* biomass showed increased peaks impacting the parent chemical of congo red, it proved to be more effective for the surface sorption of congo red compared to *A. terreus* biomass [24] concentration, however, 66 and 70% germination index was noted when exposed to the degradation product of direct blue-1 dye. As a result, treating *A. terreus* decreased the toxicity of direct blue-1 dye degradation products. As a result, *A. terreus* was employed to decolorize the direct blue-1 dye, and this study demonstrated that their metabolites were less hazardous to plant growth [88].

Garden cress (*Lepidium sativum*) seedlings were irrigated with water treated with *Phanerochaete chrysosporium* to test for toxicity. Compared to untreated plants, the fungi-treated plants exhibited a significantly higher germination rate [89]. In another investigation, *Phaseolus mungo* (black gram) and *Sorghum vulgare* (jowar) seeds were irrigated with water treated by *Marasmius* sp. after they had been contaminated with Diazo Reactive dye. For *S. vulgare*, the maximum mean shoots and root lengths were observed [90]. Ali et al. [91] examined the phytotoxicity of *A. niger* and *T. viride* on *Vigna radiata*. They found that plants treated with water remediated with *A. niger* exhibited the highest rate of germination. The study conducted by Singh and Dwivedi [24] revealed that the germination rate, root length, and shoot length of *S. leucopersicum* and *T. aestivum* were higher in the control treatment and fungal-treated congo red dye solution compared to the pure dye treatment.

## Conclusions

**Fig. 8:**
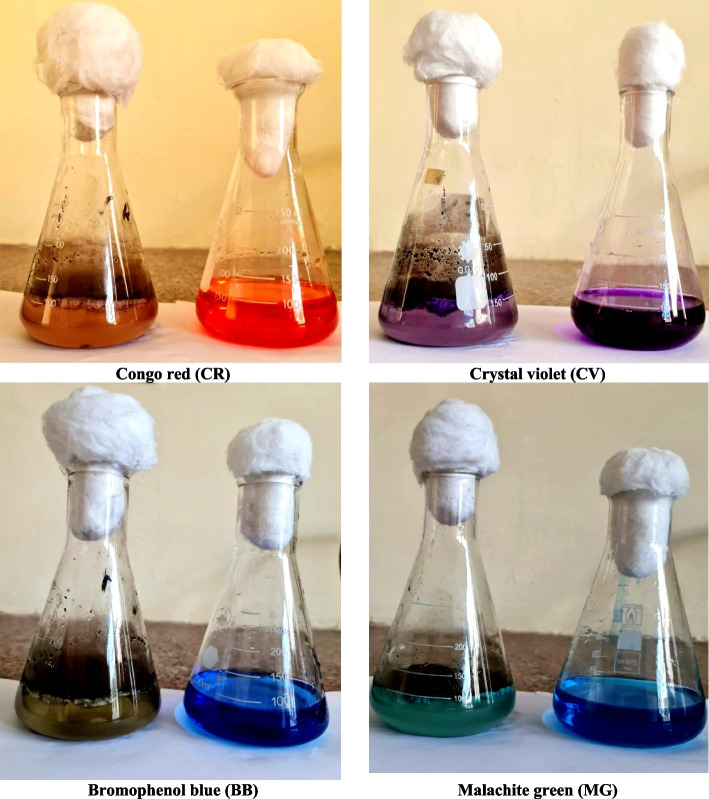
Decolonization of azo dyes by A. flavus

The utilization of fungi that efficiently decolorize and degrade synthetic dyes with diverse chemical structures is essential to the efficacy of the microbial cycle in eliminating or degrading dyes from wastewater. *A. flavus* exhibited the greatest percentage of azo dye decolorization after eight days of incubation at 30° C, pH 6, sucrose as carbon source and NaNO_3_ as nitrogen source under static conditions. Laccase, manganese peroxidase, and lignin peroxides carried out the oxidative pathway-mediated biodegradation of azo dyes, resulting in a less hazardous byproduct. In comparison to control plants, the phytotoxicity assay results indicated that the degraded product was less harmful to the growth and germination rates of maize and common bean plants. As a result, *A. flavus* may be a useful microorganism for efficiently degrading azo dyes (Fig. 8). According to our findings, the bioremediation method is the best for reducing dye toxicity because it is inexpensive and safe for the environment.

## Data Availability

All the data available in the manuscript.

## References

[1] Nachiyar CV, Rakshi AD, Sandhya S, Jebasta NBD, Nellore J. Developments in treatment technologies of dye-containing effluent: A review. Case Studies in Chemical and Environ Eng. 2023;3:100339.

[2] Solayman HM, Hossen MA, Abd Aziz A, Yahya NY, Leong KH, Sim LC, Monir MU, Zoh KD. Performance evaluation of dye wastewater treatment technologies: A review. J Environ Chemical Eng. 2023;11(3):109610.

[3] Khan MD, Singh A, Khan MZ, Tabraiz S, Sheikh J. Current perspectives, recent advancements, and efficiencies of various dye-containing wastewater treatment technologies. J Water Process Eng. 2023;53:103579.

[4] Fito J, Abewaa M, Mengistu A, Angassa K, Ambaye AD, Moyo W, Nkambule T. Adsorption of methylene blue from textile industrial wastewater using activated carbon developed from *Rumex abyssinicus* plant. Sci Rep. 2023;13(1):5427. 37012298 10.1038/s41598-023-32341-w PMC10070411

[5] Mnif I, Maktouf S, Fendri R, Kriaa M, Ellouze S, Ghribi D. Improvement of methyl orange dye biotreatment by a novel isolated strain, *Aeromonas veronii* GRI, by SPB1biosurfactant addition. J Environ Sci Pollut Res. 2016;23:1742–54. 10.1007/s11356-015-5294-9 26396008

[6] Alzain H, Kalimugogo V, Hussein K, Karkadan M. A Review of Environmental Impact of Azo Dyes. Int J Res Rev. 2023;10:64–689.

[7] Islam M, Mostafa M. Textile dyeing effluents and environment concerns - A review. J Environ Sci Nat Resour. 2019;11:131–44.

[8] Pavithra KG, Kumar PS, Jaikumar V, Rajan PS. Removal of colorants from wastewater: a review on sources and treatment strategies. J Ind Eng Chem. 2019;75:1–19.

[9] Rehman K, Shahzad T, Sahar A, Hussain S, Mahmood F, Siddique MH, Siddique MA, Rashid MI. Effect of Reactive Black 5 azo dye on soil processes related to C and N cycling. PeerJ. 2018;6:e4802. 29844965 10.7717/peerj.4802 PMC5969049

[10] Vital RK, Saibaba KVN, Shaik KB. Dye removal by adsorption: a review. J Bioremediation Biodegrad. 2016;7:371.

[11] Karimifard S, Reza M, Moghaddam A. Application of response surface methodology in physicochemical removal of dyes from wastewater: a critical review. Sci Total Environ. 2018;640–641:772–97. 30021324 10.1016/j.scitotenv.2018.05.355

[12] Yusuf M. Synthetic dyes: a threat to the environment and water ecosystem. Textiles Cloth. 2019;19:11–26.

[13] Zafar S, Bukhari DA, Rehman A. Azo dyes degradation by microorganisms – An efficient and sustainable approach. Saudi J Biol Sci. 2022;29(12):103437. 36131780 10.1016/j.sjbs.2022.103437 PMC9483650

[14] Liu T, Aniagor CO, Ejimofor MI, Menkiti MC, Wakawa YM, Li J, Akbour RA, Yap PS, Lau SY, Jeevanandam J. Recent developments in the utilization of modified graphene oxide to adsorb dyes from water: A review. J Indust Eng Chem. 2023;117:21–37.

[15] Meena H, Busi S. Biosorption of dye and heavy metal pollutants by fungal biomass: a sustainable approach. In: Prasad R, Eds., Mycoremediation and Environmental Sustainability. Fungal Biology book series. Springer, Cham. 2018; pp 253–271. 10.1007/978-3-319-77386-5_10

[16] Qin W, Guo S, Li Q, Tang A, Liu H, Liu Y. Biotransformation of the azo dye reactive orange 16 by Aspergillus flavus A5P1: Performance, genetic background, pathway, and mechanism. J Hazard Mat. 2024;468:133562. 10.1016/j.jhazmat.2024.133562 38401208

[17] Garcia-Segura S, Ocon JD, Nan M. Electrochemical oxidation remediation of real wastewater effluents — A review. Process Saf Environ Prot. 2017;113:48–67.

[18] Caicedo-Montoya C, Copete-Pertuz LS, Correa-Londono GA, Mora-Martínez AL, Yepes-Pérez M. Decolorization of colored wastewaters with Turquoise Blue dye by the *Leptosphaerulina* sp. native Colombian fungus-Influence of operational parameters. Dyna 2022; 89(221):121–131.

[19] Vikrant K, Giri BS, Raza N, Roy K, Kim KH, Rai BN, Singh RS. Recent advancements in bioremediation of dye: current status and challenges. Bioresource Technol. 2018;253:355–67. 10.1016/j.biortech.2018.01.029 29352640

[20] Upadhyay R, Przystaś W, Dave B. Myco-remediation of synthetic dyes: a comprehensive review on contaminant alleviation mechanism, kinetic study and toxicity analysis. Int J Environ Sci Technol. 2024. 10.1007/s13762-024-05793-4.

[21] Patel BY, Patel HK. Current approaches toward the removal of methylene blue dye from synthetic textile effluent using bacterial treated agricultural waste absorbent through statistical design. Heliyon. 2023;9(9):e19857. 37809607 10.1016/j.heliyon.2023.e19857 PMC10559251

[22] Ghafoor S, Ali E, Rahim F, Bukhari DA, Shafiq S, Hussain SZ, Rehman A. Evaluation of azo dyes degradation potential of Aspergillus strains: A strategy for waste management. J of Hazard Materials Advances. 2024;16:100475.

[23] Saravanan A, Kumar PS, Duc PA, Rangasamy G. Strategies for microbial bioremediation of environmental pollutants from industrial wastewater: A sustainable approach. Chemosphere. 2023;313:137323. 36410512 10.1016/j.chemosphere.2022.137323

[24] Singh G, Dwivedi SK. Mechanistic, adsorption kinetics and confirmatory study of Congo red dye removal by native fungus *Aspergillus niger*. Biomass Conv Bioref. 2022. 10.1007/s13399-022-03369-1.

[25] Yan J, Wang P, Wang L, Jin Q, Ali AS, He Y, Wang Y, Sun Y, Li A, Adwy W, Ahmed RH. Bio-decolorization of synthetic dyes by a novel endophytic fungus *Penicillium janthinellum* LM5 from blueberry pulp. Biochem Eng J. 2023;195:108909.

[26] Khan SA, Mehmood S, Shabbir SB, Ali S, Alrefaei AF, Albeshr MF, Hamayun M. Efficacy of fungi in the decolorization and detoxification of remazol brilliant blue dye in aquatic environments. Microorganisms. 2023;11(3):703. 36985276 10.3390/microorganisms11030703 PMC10058383

[27] Skanda S, Bharadwaj PSJ, Kar S, Sai Muthukumar V, Vijayakumar BS. Bioremoval capacity of recalcitrant azo dye Congo red by soil fungus Aspergillus arcoverdensis SSSIHL-01. Bioremed J. 2023;27(1):32–43.

[28] Lira Pérez J, Rodríguez VR. Removal of orange G dye by *Aspergillus niger* and its effect on organic acid production. Preparative Biochem Biotechnol. 2023;53(7):860–71. 10.1080/10826068.2022.2153368 36527445

[29] Thao TTP, Nguyen-Thi ML, Chung ND, Ooi CW, Park SM, Lan TT, Quang HT, Khoo KS, Show PL, Huy ND. Microbial biodegradation of recalcitrant synthetic dyes from textile-enriched wastewater by *Fusarium oxysporum*. Chemosphere. 2023;325:138392. 36921772 10.1016/j.chemosphere.2023.138392

[30] Herath IS, Udayanga D, Jayasanka DJ, Hewawasam C. Textile dye decolorization by white rot fungi–A review. Biores Technol Rep. 2024;25:101687.

[31] Alam R, Mahmood RA, Islam S, Ardiati FC, Solihat NN, Alam MB, Lee SH, Yanto DHY, Kim S. Understanding the biodegradation pathways of azo dyes by immobilized white-rot fungus, *Trametes hirsuta* D7, using UPLC-PDA-FTICR MS supported by in silico simulations and toxicity assessment. Chemosphere. 2023;313:137505. 36509189 10.1016/j.chemosphere.2022.137505

[32] Jeyabalan J, Veluchamy A, Kumar A, Chandrasekar R, Narayanasamy S. A review on the laccase assisted decolourization of dyes: Recent trends and research progress. J Taiwan Instit Chem Eng. 2023;151:105081.

[33] Chau TP, Rajkumar R, Aloufi AS, Krishnan R, Tharifkhan SA. Textile effluents decolourization potential of metal tolerant Aspergillus species and optimization of biomass concentration and temperature. Environ Rese. 2023;2023(232):116294. 10.1016/j.envres.2023.116294 37268209

[34] Rauf MA, Salman-Ashraf S. Survey of recent trends in biochemically assisted degradation of dyes. Chem Eng J. 2012;209:520–30.

[35] Przystas W, Zablocka-Godlewska E, Grabinska-Sota E. Efficacy of fungal decolorization of a mixture of dyes belonging to different classes. Brazil J Microbiol. 2015;46:415–24. 10.1590/S1517-838246246220140167 PMC4507533 26273256

[36] El Awady ME, El-Shall FN, Mohamed GE, Abd-Elaziz AM, Abdel-Monem MO, Hassan MG. Exploring the decolorization efficiency and biodegradation mechanisms of different functional textile azo dyes by *Streptomyces albidoflavus* 3MGH. BMC Microbiol. 2024;24(1):210. 38877404 10.1186/s12866-024-03347-9 PMC11179346

[37] Jonstrup M, Kumar N, Guieysse B, Murto M, Mattiasson B. Decolorization of textile dyes by Bjerkandera sp. BOL 13 using waste biomass as carbon source. J Chem Technol Biotechnol. 2013; 88(3): 388–394.

[38] Beni AA, Esmaeili A. Biosorption, an efficient method for removing heavy metals from industrial effluents: a review. Environ Technol Innov. 2020;17:100503.

[39] Rangabhashiyam S, Suganya E, Selvaraju N, Varghese LA. Significance of exploiting non-living biomaterials for the biosorption of wastewater pollutants. World J Microbio Biotechnol. 2014;30:1669–89. 10.1007/s11274-014-1599-y 24436063

[40] Adeniyi AG, Ighalo JO. Biosorption of pollutants by plant leaves: an empirical review. J Environ Chem Eng. 2019;7:103100.

[41] Shah H, Yusof F, Alam MZ. A new technique to estimate percentage decolorization of synthetic dyes on solid media by extracellular laccase from white-rot fungus. Bioremed J. 2021;27(1):66–74.

[42] Srinu A, Vijaya LD, Murali S, Prasad DV. Decolorization of anthraquinone dyes by *Aspergillus* strains and also optimization of lignolytic enzymes. Int J Rec Sci Res. 2017;8(7):18547–53.

[43] Liu S, Xu X, Kang Y, Xiao Y, Liu H. Degradation and detoxification of azo dyes with recombinant ligninolytic enzymes from Aspergillus sp. with secretory overexpression in *Pichia pastoris*. Royal Society Open Sci. 2020;7(9):200688. 10.1098/rsos.200688 PMC7540776 33047030

[44] Lowry OH, Rosebrough NJ, Farr AL, Randall RJ. Protein measurement with the Folin phenol reagent. J Biol Chem. 1951;193(1):265–75. 14907713

[45] Velikova V, Yordanov I, Edreva AJ. Oxidative stress and some antioxidant systems in acid rain-treated bean plants: protective role of exogenous polyamines. Plant Sci. 2000;151(1):59–66.

[46] Ahmed F, Dewani R, Pervez MK, Mahboob SJ, Soomro SA. Non-destructive FT-IR analysis of mono azo dyes. Bulg Chem Commun. 2016;48(1):71–7.

[47] Sadeghi H, Khazaei F, Yari L, Sheidaei S. Effect of seed osmopriming on seed germination behavior and vigor of soybean (*Glycine max* L.). ARPN J Agric Biol Sci. 2011;6(1):39–43.

[48] Barik SR, Pandit E, Sanghamitra P, Mohanty SP, Behera A, Mishra J, Nayak DK, Bastia R, Moharana A, Sahoo A, Pradhan SK. Unraveling the genomic regions controlling the seed vigour index, root growth parameters and germination percent in rice. PLoS ONE. 2022;17(7):e0267303. 35881571 10.1371/journal.pone.0267303 PMC9321372

[49] Shabir M, Yasin M, Hussain M, Shafiq I, Akhter P, Nizami A, Jeon B, Park Y. A review on recent advances in the treatment of dye-polluted wastewater. J Indust Eng Chem. 2022;112:1–19.

[50] Hefnawy MA, Gharieb MM, Shaaban MT, Soliman AM. Optimization of culture condition for enhanced decolorization of direct blue dye by *Aspergillus flavus* and *Penicillium canescens*. JApp Pharmace Sci. 2017;7(2):083–92.

[51] Singh L, Singh P. Biodegradation of Textile Dyes, Bromophenol Blue and Congo red by fungus *Aspergillus flavus*. Environ We Int J Sci Tech. 2010;5:235–42.

[52] Kunjadia P, Patel FD, Nagee A, Mukhopadhyaya NP, Dave SG. Crystal Violet (Triphenyl Methane Dye) Decolourization potential of *Pleurotus ostreatus* (MTCC 142). Bio Reso. 2012;7:1189–99.

[53] Cheng ZiZhang CZ, Yang ZhouPing YZ, Hu Rong HR, Jing DeJun JD, Chen HuaPing CH. Decolorization of 12 kinds of dyes by the mycelium pellets of Trametesgallica under non-sterile condition. Mycosystema. 2012;31(6):878–89.

[54] Yang B, Feng LD, Zhang LY. Decolorization of wastewater containing reactive brilliant blue dyes by laccase. Chin J Environ Eng. 2012;6(10):3514–8.

[55] Sheam MM, Biswas SK, Ahmed KR, Syed S, Bin Hossain MS, Khan MSA, Hasan MR, Zohra FT, Rahman MM. Mycoremediation of reactive red HE7B dye by *Aspergillus salinarus* isolated from textile effluents. Curr Res Microb Sci. 2021;2: 100056. 34841347 10.1016/j.crmicr.2021.100056 PMC8610306

[56] Hashem RA, Samir R, Essam TM, Ali AE, Amin MA. Optimization and enhancement of textile reactive Remazol black B decolorization and detoxification by environmentally isolated pH tolerant *Pseudomonas aeruginosa* KY284155. AMB Expr. 2018;8:83. 10.1186/s13568-018-0616-1 PMC5962525 29785517

[57] Namdhari BS, Rohilla SK, Salar RK, Gahlawat SK, Bansal P, Saran AK. Decolorization of reactive blue MR, using Aspergillus species isolated from textile waste water. ISCA J Biol Sci. 2012;1(1):24–9.

[58] Fetyan NA, Abdelazeiz AZ, Ismail IM, Shaban SA. Oxidative decolorization of direct blue 71 azo dye by Saccharomyces cerevisiae catalyzed by nano zero-valent iron. Ann Res Rev Biol. 2016;11(2):1–12.

[59] Asses N, Ayed L, Hkiri N, Hamdi M. Congo red decolorization and detoxification by *Aspergillus niger*: removal mechanisms and dye degradation pathway. Int J Biomed Res. 2018;7:1–9. 10.1155/2018/3049686 PMC6106729 30175122

[60] Arunprasath T, Sudalai S, Meenatchi RS, Jeyavishnu K, Arumugam A. Biodegradation of triphenyl methane dye malachite green by a newly isolated fungus strain. Biocatal Agric Biotechnol. 2019;17:672–9.

[61] Ning C, Qingyun L, Aixing T, Wei S, Youyan L. Decolorization of a variety of dyes by Aspergillus flavus A5p1. Bioprocess Biosyst Engine. 201841:511–8. 10.1007/s00449-017-1885-9 29299675

[62] Binupriya AR, Sathishkumar M, Swaminathan K, Kuz CS, Yun SE. Comparative studies on removal of Congo red by native and modified mycelial pellets of *Trametes versicolor* in various reactor modes. Biores Technol. 2008;99:1080–8. 10.1016/j.biortech.2007.02.022 17416520

[63] Kalyani P, Hemalatha V, Vineela K, Hemalatha KPJ. Degradation of Toxic Dyes- A Review. Int J Pure App Biosci. 2016;4(5):81–9.

[64] Ameen F, Dawoud TM, Alshehrei F, Alsamhary K, Almansob A. Decolorization of acid Blue 29, disperse Red 1 and Congo Red by different indigenous fungal strains. Chemosphere. 2021;271:129532. 33429264 10.1016/j.chemosphere.2021.129532

[65] Singh G, Dwivedi SK. Decolorization and degradation of Direct Blue-1 (Azo dye) by newly isolated fungus *Aspergillus terreus* GS28, from sludge of carpet industry. Environ Technol Innovat. 2020;18:100751.

[66] Kaushik G, Seth R. Molecular Characterization of Dye Degrading *Aspergillus flavus* Strain Gkrs09 and FT-IR Analysis of The Degraded Products. J Pharmace. 2023;8:3195–205.

[67] Saratale RG, Saratale GD, Chang JS, Govindwar SP. Bacterial decolorization and degradation of azo dyes: A review. J Taiwan Inst Chem Eng. 2011;42(1):138–57.

[68] Pawłowska J, Okrasińska A, Kisło K, Aleksandrzak-Piekarczyk T, Szatraj K, Dolatabadi S, Muszewsk A. Carbon assimilation profiles of mucoralean fungi show their metabolic versatility. Sci Rep. 2019;9:11864. 31413281 10.1038/s41598-019-48296-w PMC6694110

[69] Solis M, Solis A, Pérez HI, Manjarrez N, Flores M. Microbial decolouration of azo dyes: A review. Process Biochem. 2012;47:1723–48.

[70] Hou B, Hu Y, Sun J. Performance and microbial diversity of microbial fuel cells coupled with different cathode types during simultaneous azo dye decolorization and electricity generation. Bioresour Technol. 2012;111:105–10. 22386629 10.1016/j.biortech.2012.02.017

[71] Sweety, Vats S, Kumar M, Kumar V, Gupta S, Garg SK. Nitrogen and Carbon Sources Influencing Mycoremediation of Textile Dyes Using Novel Autochthonous Fungal Isolates. Anal Chem. Lett. 2017; 7(5): 632–646

[72] Bettin F, Cousseau F, Martins K, Zaccaria S, Girardi V, Silveira MM, Dillon AJ. Effects of pH, temperature and agitation on the decolourisation of dyes by Laccase containing enzyme preparation from *Pleurotussajor-caju*. Braz Arch Biol Technol. 2019;62:19180338.

[73] Bankole OP, Adekunle AA, Govindwar SP. Enhanced decolorization and biodegradation of Acid Red 88 dye by newly isolated fungus. Achaetomium strumarium J Environ Chem Eng. 2018;6:1589–600.

[74] Pandi A, Kamini NR, Saravanan P, Gowthaman MK. A sustainable approach for degredation of leather dyes by a new fungal laccase. J Clean Prod. 2018;211:590–7.

[75] Mani P, Keshavarz T, Chandra TS, Kyazze G. Decolourization of acid orange 7 in a microbial fuel cell with a laccase-based biocathode: Influence of mitigating ph changes in the cathode chamber. Enzyme Microb Technol. 2017;96:170–6. 27871379 10.1016/j.enzmictec.2016.10.012

[76] Kang Y, Xu X, Pan H, Tian J, Tang W, Liu S. Decolorization of mordant yellow 1 using *Aspergillus* sp. TS-A CGMCC 12964 by biosorption and biodegradation. Bioengineered. 2018; 9(1):222–32. 10.1080/21655979.2018.1472465 PMC6984770 29991323

[77] Kabra AN, Khandare RV, Govindwar SP. Development of a bioreactor for remediation of textile effluent and dye mixture: a plant-bacterial synergistic strategy. Water Res. 2013;47(3):1035–48. 23245543 10.1016/j.watres.2012.11.007

[78] Sinha A, Osborne WJ. Biodegradation of reactive green dye (RGD) by indigenous fungal strain VITAF-1. Int J Biodeterior Biodegrad. 2016;114:176–83.

[79] Shanmugam S, Ulaganathan P, Swaminathan K, Sadhasivam S, Wu YR. Enhanced biodegradation and detoxification of malachite green by *Trichoderma asperellum* laccase: degradation pathway and product analysis. Int Biodeterior Biodegrad. 2017;125:258–68.

[80] Wang N, Chu Y, Wu F, Zhao Z, Xu X. Decolorization and degradation of congo red by a newly isolated White rot fungus, *Ceriporia lacerate,* from decayed mulberry branches. Int J Biodetor Biodegrad. 2017;117:236–44.

[81] Barapatre A, Aadil KR, Jha H. Biodegradation of malachite green by the ligninolytic fungus Aspergillus flavus. Clean: Soil, Air, Water. 2017;45(4):1600045.

[82] Zhang X, Wang M, Lin L, Xiao G, Tang Z, Zhu X. Synthesis of novel laccase-biotitania biocatalysts for malachite green decolorization. J Biosci Bioeng. 2018;126(1):69–77. 29567373 10.1016/j.jbiosc.2018.01.021

[83] Singh G, Dwivedi SK. Biosorptive and Biodegradative Mechanistic Approach for the Decolorization of Congo Red Dye by *Aspergillus* Species. Bull Environ Contam Toxicol. 2022;108:457–67. 34625833 10.1007/s00128-021-03380-8

[84] Chakraborty S, Basak B, Dutta S, Bhunia B, Dey A. Decolorization and biodegradation of congo red dye by a novel white rot fungus *Alternaria alternata* CMERI F6. Bioresour Technol. 2013;147:662–6. 24034987 10.1016/j.biortech.2013.08.117

[85] Kumar V, Dwivedi SK. Hexavalent chromium reduction ability and bioremediation potential of *Aspergillus flavus* CR500 isolated from electroplating wastewater. Chemosphere. 2019; 237:10.1016/j.chemosphere.2019.124567 31549665

[86] Anbarani MZ, Nourbakhsh S, Toolabi A, Bonyadi Z. Biodegradation of crystal violet dye by *Saccharomyces cerevisiae* in aqueous medium. Heliyon. 2023;9(9):e19460. 37810043 10.1016/j.heliyon.2023.e19460 PMC10558598

[87] Salem SS, Mohamed A, El-Gamal M, Talat M, Fouda A. Biological decolorization and degradation of azo dyes from textile wastewater effluent by *Aspergillus niger*. Egyptian J Chem. 2019;62(10):1799–813.

[88] Nouren S, Bhatti HN. Mechanistic study of degradation of basic violet 3 by Citrus limon peroxidase and phytotoxicity assessment of its degradation products. Biochem Eng J. 2015;95:9–19.

[89] Almeida EJR, Corso CR. Comparative study of toxicity of azo dye Procion Red MX-5B following biosorption and biodegradation treatments with the fungi *Aspergillus niger* and *Aspergillus terreus*. Chemosphere. 2014;112:317–22. 25048922 10.1016/j.chemosphere.2014.04.060

[90] Vantamuri AB, Shettar AK. Biodegradation of Diazo Reactive dye (Green HE4BD) by *Marasmius* sp. BBKAV79. Chem Data Collect. 2020; 28: 1–9.

[91] Ali E, Amjad I, Rehman A. Evaluation of azo dyes degradation potential of fungal strains and their role in wastewater treatment. Saudi J Biolog Sci. 2023;30(8):103734. 10.1016/j.sjbs.2023.103734 PMC10359666 37483839

